# Supplementary material for: Shoulder Magnetic Resonance Arthrography with the Internal and External Rotation Positions of the Humeral Head in the Evaluation of SLAP Lesions
Source: Diagnostics (Basel). 2022 Sep 15;12(9):2230. doi: 10.3390/diagnostics12092230 (PMC9497654; doi:10.3390/diagnostics12092230)
Supplement: Supplementary file 1 [file diagnostics-12-02230-s001.zip › diagnostics-1855226-supplementary.pdf]

**Table S1.** MRA acquisition protocol.

| | Acquisition Plane | Fat saturation | Arm Position | Voxel Size (mm) | FoV read (mm)/Phase (%) | TR/TE (ms) | Slice Thickness (mm) | Distance Factor (%) | Averages/ Concatenations |
|---|---|---|---|---|---|---|---|---|---|
| T1 tse | Axial | No | N | 0.8x0.8x3 | 250/84.4 | 600/11 | 3 | 30 | 2/2 |
| T1 tse | Coronal | No | N | 0.7x0.7x3 | 230/100 | 709/9.7 | 3 | 30 | 1/2 |
| T1 tse | Sagittal | No | N | 1x1x3 | 250/84.4 | 568/9.4 | 3 | 30 | 1/2 |
| PD tse | Coronal | Yes | N | 0.4x0.4x3 | 200/100 | 3000/27 | 3 | 20 | 2/1 |
| T1 vibe | Coronal | No | N | 0.8x0.8x0.9 | 160/100 | 16.3/7.16 | 0.9 | 20 | 1/1 |
| T1 tse fast | Axial | No | IR | 1x1x3.5 | 250/84.4 | 400/9.4 | 3.5 | 30 | 1/2 |
| T1 tse fast | Axial | No | ER | 1x1x3.5 | 250/84.4 | 400/9.4 | 3.5 | 30 | 1/2 |

Note – N = neutral; IR = internal rotation; ER = external rotation.
